# Left Ventricular Thrombosis Secondary to Severe Myocardial Contusion Without Coronary Artery Injury Following Blunt Injury: A Case Report

**DOI:** 10.3390/jcm15093293

**Published:** 2026-04-25

**Authors:** Yo Huh, Jonghwan Moon

**Affiliations:** 1 Department of Trauma and Acute Care, Ajou University School of Medicine, Suwon 16499, Republic of Korea; ermdhuhyo@gmail.com; 2 Gyeonggi Southern Regional Trauma Center, Ajou University Hospital, Suwon 16499, Republic of Korea

**Keywords:** blunt trauma, myocardial contusion, thrombosis, ventricular dysfunction

## Abstract

**Background:** Left ventricular (LV) thrombosis after blunt trauma is uncommon and is most often attributed to traumatic coronary artery injury; however, it can also arise from severe myocardial contusions. Here, we report a case of LV thrombosis due to severe myocardial contusion without coronary artery injury. **Case Presentation:** A 36-year-old man struck by industrial fan fragments presented with hemorrhagic shock. Focused Assessment with Sonography for Trauma revealed cardiac tamponade. An emergent sternotomy was performed under cardiopulmonary bypass via the femoral vessels, which exposed severe contusion-associated hemorrhage with epicardial–myocardial dissection at the LV apex. On postoperative day (POD) 5, transthoracic echocardiography showed apical akinesia with mural thrombi; prophylactic anticoagulation was escalated and later transitioned to warfarin. Coronary computed tomography on POD 21 and invasive angiography at 6 months revealed negative findings. The thrombi resolved within 3 months; however, apical akinesia persisted. After discontinuing anticoagulation, a transient ischemic event occurring at 9 months prompted direct oral anticoagulant therapy. Apical akinesia persisted for over 7 years without recurrent thrombosis. **Conclusions:** This case underscores the importance of vigilance for intracardiac thrombosis in severe contusions, as well as the value of stepwise imaging (contrast echocardiography) and cautious, individualized discontinuation of anticoagulation when regional dysfunction persists.

## 1. Introduction

Blunt cardiac injury (BCI) encompasses a wide spectrum of conditions ranging from clinically silent myocardial contusions to fatal chamber ruptures. Myocardial contusion is the most prevalent form of BCI, with clinical manifestations ranging from asymptomatic arrhythmias to severe hemodynamic compromise, cardiogenic shock, and cardiac rupture in rare cases [1].

Left ventricular (LV) thrombosis is a classic complication of myocardial infarction, most often associated with coronary artery disease and ischemic injury [2]. Intracardiac thrombosis has been described in trauma but is typically secondary to traumatic coronary artery injury with occlusion [3]. In contrast, LV thrombus formation after myocardial contusion alone, without evidence of coronary injury or infarction, is rare and likely underrecognized in the BCI literature.

Here, we aimed to report a case of LV thrombosis following blunt myocardial contusion without angiographic or clinical evidence of coronary artery injury and highlight the diagnostic considerations and management trade-offs relevant to trauma care.

## 2. Case Report

### 2.1. Initial Presentation and Assessment

**Figure 1:**
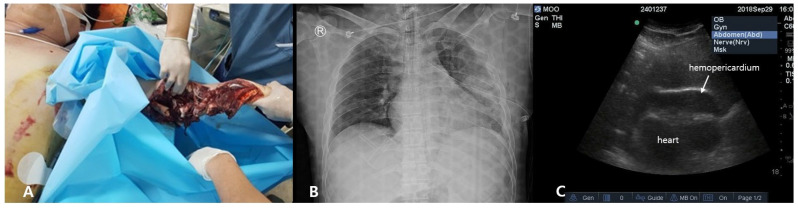
Initial presentation. (**A**) Mangled left arm. (**B**) Initial chest radiograph. (**C**) FAST showing hemopericardium.

A 36-year-old man was struck on the arm and torso by fragments from a fan while working at a power plant. He was transported to the trauma center by helicopter. Upon arrival, the emergency medical service paramedics had already established an intravenous line and were administering oxygen through a 15 L oxygen non-rebreather mask. His initial vital signs were as follows: blood pressure, 70/52 mmHg; heart rate, 121 beats/min; and oxygen saturation, 100%. The initial Glasgow Coma Scale score was 12 (E3, V4, and M5). A massive transfusion protocol was initiated 2 min after arrival, and intubation was performed. Decreased breath sounds were noted on the left chest with flail motion; a chest tube was inserted simultaneously. The left arm showed a severe mangled injury distal to the midshaft of the humerus, and compression and a tourniquet were applied. Focused Assessment with Sonography for Trauma demonstrated cardiac tamponade with associated hemopericardium (Figure 1), and an emergency operation was performed approximately 50 min after arrival.

### 2.2. Operation

**Figure 2:**
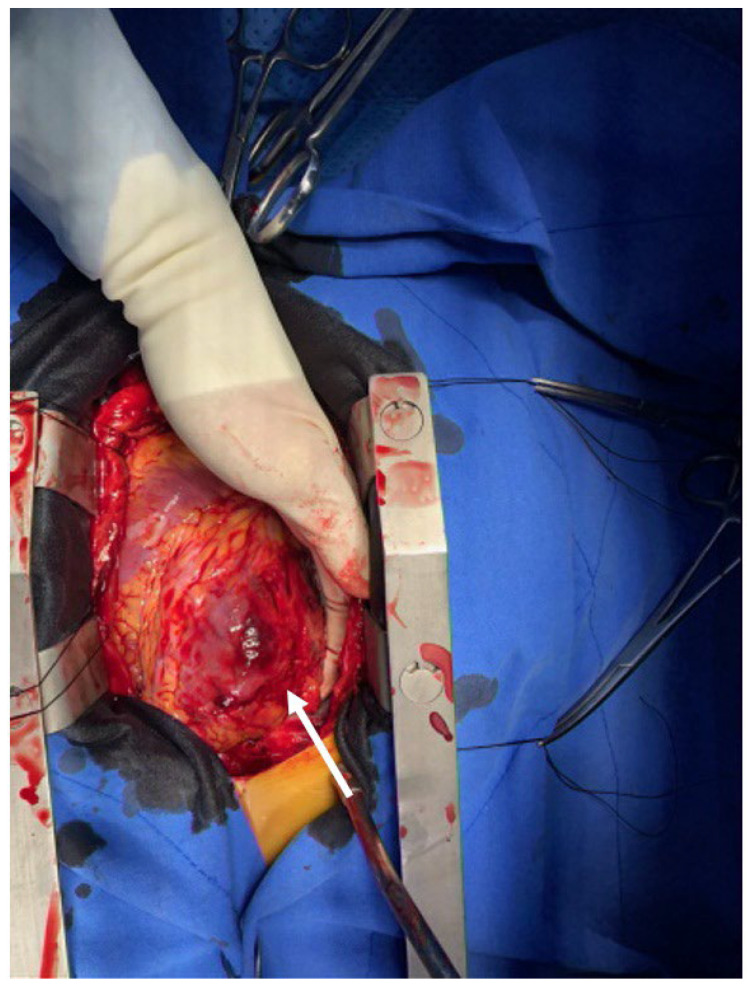
Intraoperative field image. Intraoperative photograph after median sternotomy and pericardiotomy showing severe contusion injury of the left ventricular (LV) apex and anterolateral wall. The arrow indicates a subepicardial hematoma with epicardial–myocardial delamination; no full-thickness rupture or active bleeding was identified.

The patient remained in persistent shock with a large hemopericardium, for which cardiopulmonary bypass was performed via the left femoral artery and vein. Median sternotomy revealed a massive hemopericardium. The LV apex and mid-anterolateral wall exhibited severe contusion-associated hemorrhage accompanied by a hematoma (Figure 2) and dissection between the epicardium and myocardium. However, no active bleeding was identified. Hemostasis was achieved, and reinforcement was performed using TachoSil and surgical glue. Concomitantly, the orthopedic surgery team performed left elbow amputation. Although spontaneous healing of a true LV free-wall rupture is unlikely, the lesion in this case may have been temporarily contained by intramyocardial dissection and/or an epicardial hematoma, resulting in a sealed or contained rupture rather than free perforation.

### 2.3. Postoperative Course

**Figure 3:**
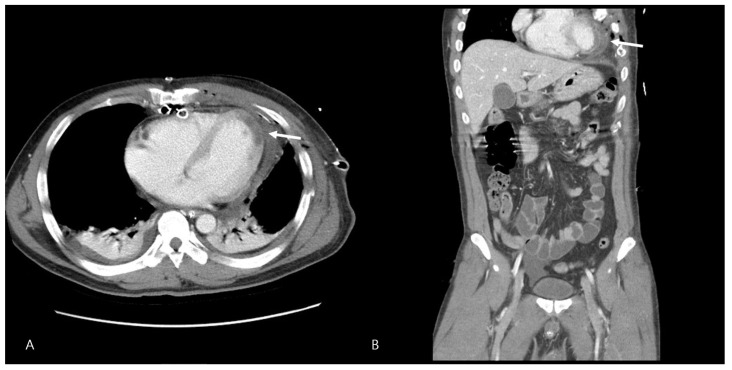
Essential images of chest CT findings. Postoperative day 1 contrast-enhanced chest CT. Axial (**A**) and coronal reformatted (**B**) images show a focal hypoattenuating area in the anterior/anterolateral wall of the left ventricle (arrows), consistent with myocardial contusion.

The patient’s postoperative course was initially stable, with the cessation of bleeding and normalization of vital signs. Chest computed tomography (CT) performed on postoperative day (POD) 1 revealed severe myocardial contusion involving the apex and anterior wall of the LV (Figure 3).

Serial assessment demonstrated marked but incomplete recovery of myocardial injury. Initial cardiac biomarkers were markedly elevated, with CK 569 U/L, CK-MB 55.7 μg/L, and troponin I 55.7 ng/mL. Cardiac enzyme levels showed a gradual decline from 3 days later.

Serial electrocardiographic follow-up demonstrated temporal evolution of myocardial injury. On admission, the initial ECG showed sinus tachycardia with marked anterolateral ST-T abnormalities. At 1 month after injury, sinus rhythm was restored, but poor R-wave progression, persistent anterior Q-wave abnormalities, and evolving T-wave inversions in the inferior and anterolateral leads remained. At 3 months after injury, poor R-wave progression and anterior Q-wave abnormalities persisted, with residual lateral T-wave changes.

**Figure 4:**
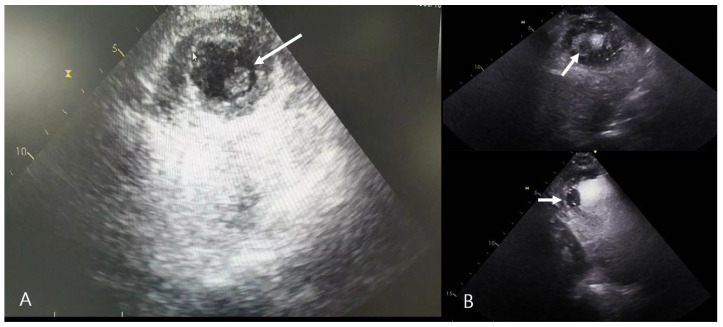
Postoperative echocardiography demonstrates mural thrombi in the left ventricular apex. (**A**) Transthoracic echocardiography shows an echogenic mass adherent to the left ventricular apex (arrow). (**B**) Contrast-enhanced echocardiography delineates a non-opacified apical filling defect (arrows), confirming mural thrombosis.

On POD 5, transthoracic echocardiography (TTE) revealed regional wall motion akinesia at the LV apex, accompanied by mural thrombi measuring 1.9 × 0.9 cm and 2.5 × 1.2 cm. The prophylactic dose of enoxaparin that had been administered to prevent venous thromboembolism was subsequently escalated to a therapeutic anticoagulation regimen. Follow-up contrast echocardiography confirmed a filling defect at the LV apex consistent with a thrombus, measuring 1.2 × 1.4 cm (Figure 4).

The patient was transferred to the general ward on POD 6, and oral warfarin therapy was initiated, targeting an international normalized ratio (INR) of 2.0–3.0, because evidence for the use of direct oral anticoagulants in LV thrombosis was limited at that time and warfarin remained the recommended treatment.

Postoperatively, the patient required intensive pulmonary care, including mechanical ventilation, due to flail chest, shock-related atelectasis, and pulmonary edema. After weaning from mechanical ventilation, further evaluation was difficult because of poor cooperation associated with delirium. Sedation for diagnostic testing was considered, but was judged to pose a high risk. As there were no signs of ongoing acute myocardial infarction, such as chest pain, coronary CT angiography was delayed until the patient’s condition became more stable. Coronary CT angiography performed on POD 21 revealed no evidence of coronary artery pathology. On POD 23, repeat echocardiography demonstrated a reduction in thrombus size to 5 mm; however, regional akinesia at the apex persisted with a reduced ejection fraction of 35%. Angiotensin-converting enzyme inhibitors and beta-blockers were added to optimize medical therapy. The patient continued anticoagulation therapy with warfarin and was discharged on POD 30 with New York Heart Association (NYHA) functional class III symptoms.

At the 3-month follow-up, echocardiography revealed complete resolution of the thrombus, although LV systolic dysfunction persisted, with a modest improvement in ejection fraction to 40% and the patient’s symptoms improved to New York Heart Association (NYHA) functional class II. Furthermore, 6 months after discharge, coronary angiography showed no significant abnormalities, and repeat echocardiography confirmed the absence of residual LV thrombus. By that time, the patient’s symptoms had further improved to NYHA functional class I. In line with the recommendation for 6 months of anticoagulation in patients with LV thrombosis after acute myocardial infarction, anticoagulation was discontinued.

However, at 9 months post-injury, the patient developed transient hemianopsia due to an embolic cerebrovascular event. In the absence of other identifiable risk factors and with sinus rhythm documented on ECG and no evidence of inherited or acquired thrombophilia, the event was considered likely secondary to LV thrombosis. Anticoagulation was therefore resumed with a direct oral anticoagulant (DOAC) because of poor adherence to warfarin therapy.

At 1 year, echocardiography showed an ejection fraction of 41%, which remained stable thereafter. The patient has, since, been followed up in the outpatient clinic for 7 years, with persistent apical akinesia in the left anterior descending artery territory, but no recurrent thrombosis. He remains on DOAC therapy and is currently under outpatient follow-up without significant change.

## 3. Discussion

Myocardial contusion is the most common cardiac manifestation of blunt chest trauma, but its true incidence remains uncertain because no single definitive diagnostic test or uniform case definition exists. Reported rates of blunt cardiac injury (BCI) range from 8% to 76%, largely reflecting differences in screening strategies and diagnostic criteria rather than true epidemiologic variation [1]. Clinically, myocardial contusion ranges from asymptomatic biomarker elevation or transient dysrhythmias to regional wall motion abnormalities and, rarely, hemodynamically significant pump failure. Because symptoms are often nonspecific and confounded by associated thoracic injuries, the diagnosis is generally based on the combination of mechanism, ECG findings, cardiac biomarkers, and imaging rather than on any single test [4].

Severe myocardial contusion is suggested by sustained hypotension or cardiogenic shock, malignant arrhythmias or high-grade conduction disturbances, significant LV dysfunction, or structural cardiac injury. In a prospective cohort frequently cited in practice guidance, clinically significant BCI was identified in approximately 13% of screened patients [5]. Severe myocardial contusion may present with sustained arrhythmias, hemodynamic compromise, or significant LV dysfunction. Although many rhythm disturbances are transient, delayed conduction abnormalities can occur because of scar-related remodeling. Regional wall motion abnormalities may also promote mural thrombus formation and thromboembolism, highlighting the importance of targeted echocardiographic evaluation [6]. In the most severe cases, blunt cardiac trauma may result in valvular or myocardial rupture, underscoring the need for careful surveillance after high-energy chest trauma. Current guidelines recommend initial evaluation with a 12-lead ECG and cardiac troponin in patients with suspected BCI; normal findings on both make clinically significant contusion unlikely [5]. When abnormalities are present, serial testing and targeted imaging are warranted [7]. TTE is the first-line imaging modality for assessing pericardial effusion and ventricular dysfunction, whereas CMR may be useful when echocardiographic findings are inconclusive [8].

After blunt chest trauma, intracardiac thrombosis most often reflects Virchow’s triad: (i) endothelial or endocardial injury from contusion, (ii) regional wall motion abnormalities causing stasis (classically apical akinesis or dyskinesis), and (iii) a trauma-related hypercoagulable state. The LV is the usual site, and embolic events (stroke or peripheral embolism) are recognized complications [3,4,9]. In our case, the initial surgical findings of hemopericardium with severe contusion-associated hemorrhage and epicardial–myocardial dissection at the LV apex suggested a potent substrate for thrombosis (endocardial injury + regional stasis), reinforcing Virchow’s triad in traumatic settings.

A distinct pathway is secondary ischemic thrombus formation when blunt trauma causes coronary artery injury. Most commonly, this injury involves left anterior descending or right coronary artery dissection or intimal tear, occasionally involving the left main artery, leading to regional infarction and subsequent mural LV thrombus formation [10]. Coronary thrombosis without angiographically apparent dissection has also been reported after blunt trauma (confirmed using intravascular imaging), emphasizing that primary thrombosis may follow intimal injury that is not evident on initial angiography [11]. Although less common, LV thrombus can form solely from myocardial contusion without angiographic coronary injury (as in our case) presumably due to localized myocardial hemorrhage and stunning (often apical) that create a nidus for thrombosis in a hypercoagulable milieu. Several case reports have described LV apical mural thrombi formation, with or without distal embolization, despite angiographically normal coronaries [3,12]. In our case, although secondary ischemia due to traumatic coronary artery injury or disseminated intravascular coagulation cannot be definitively excluded because coronary CT was not performed immediately after the injury, the initial operative findings, the transmural apical myocardial injury observed on the initial chest CT, and the absence of significant abnormalities on subsequent coronary CT and invasive angiography collectively support the interpretation that the LV thrombus was more likely attributable to myocardial contusion than to coronary occlusion.

Consistent with prior reports, LV thrombus after blunt trauma may develop within days of injury or emerge later, despite initially unrevealing studies. In our case, LV was first identified on postoperative day 5, highlighting the need for early screening and ongoing interval imaging when regional wall motion abnormalities persist. Delayed presentations also occur, and new ischemic features (such as angina or rising biomarkers) after initially “negative” coronary studies should prompt re-evaluation and, when appropriate, revascularization alongside thrombus-directed therapy [13].

Diagnostic imaging should be optimized for thrombus detection. Contrast-enhanced TTE increases sensitivity, and CMR with late gadolinium enhancement remains the reference standard and is preferred when echocardiography is equivocal or the apex is poorly visualized [9,14]. In our case, the diagnosis of LV thrombus by TTE was facilitated by prior knowledge of the apical injury based on the operative and CT findings.

Treatment must balance the embolization risk (stroke or systemic emboli) with the hemorrhagic risk, which is often increased in patients with trauma. Under hemostatically acceptable circumstances, most expert guidelines support systemic anticoagulation initiated with a titratable parenteral agent (such as unfractionated heparin) and transitioned to oral therapy. The American Heart Association scientific statement recommends oral anticoagulation for approximately 3 months after LV thrombus (post-MI data) with repeat imaging to verify resolution. In non-ischemic contexts, many clinicians individualize oral anticoagulation to 3–6 months depending on the recovery of LV function and bleeding risk. At the time this case occurred, evidence for DOAC use in LV thrombus was limited, and vitamin K antagonists were the recommended standard treatment. Moreover, because no specific guidance existed for traumatic LV thrombosis, treatment was guided by recommendations for LV thrombus after MI. Although more recent evidence suggests that DOACs may be a reasonable alternative to warfarin, with comparable thrombus resolution and no clear excess in embolic or major bleeding events, this was not yet sufficiently established when our patient was treated. When anticoagulation is contraindicated or unsuccessful and the thrombus is large or mobile with an imminent embolic risk, surgical or percutaneous thrombectomy may be considered on a case-by-case basis [9]. In this case, the LV thrombus had resolved by 3 months. However, the apical wall akinesia persisted. After discontinuing anticoagulation, the patient developed an ischemic event, prompting the reinstitution of therapy. These findings emphasize that if regional LV akinesia and, thus, stasis persist, anticoagulation therapy should be discontinued cautiously and be individualized. Ideally, the discontinuation should be guided by interval imaging and the evolving bleeding risk.

## 4. Conclusions

We reported a rare case of LV thrombosis arising from a severe myocardial contusion without coronary artery injury. When severe myocardial contusion is suspected after blunt trauma, clinicians should maintain a high index of suspicion for intracardiac thrombosis and promptly obtain appropriate imaging, starting with TTE (with contrast when needed) and escalating to CMR imaging if diagnostic uncertainty remains. In our case, anticoagulation was discontinued after thrombus resolution, but a subsequent embolic ischemic event occurred while apical akinesia persisted. This clinical course suggests that persistent regional LV akinesia may continue to confer thromboembolic risk even after apparent thrombus resolution. Therefore, discontinuation of anticoagulation should be approached cautiously and individualized according to interval imaging findings and bleeding risk.

## Data Availability

The raw data supporting the conclusions of this article will be made available by the authors on request.

## Abbreviations

The following abbreviations are used in this manuscript:

BCI	Blunt cardiac injury
CMR	Cardiac magnetic resonance
CT	Computed tomography
DOAC	Direct oral anticoagulant
ECG	Electrocardiography
LV	Left ventricular
POD	Postoperative day
TEE	Transesophageal echocardiography
TTE	Transthoracic echocardiography
